# Room Temperature Hydrogen Atom Scattering Experiments Are Not a Sufficient Benchmark to Validate Electronic Friction Theory

**DOI:** 10.1021/acs.jpclett.4c02468

**Published:** 2024-12-13

**Authors:** Connor L. Box, Nils Hertl, Wojciech G. Stark, Reinhard J. Maurer

**Affiliations:** † Department of Chemistry, University of Warwick, Gibbet Hill Road, CV4 7AL Coventry, U.K.; ‡ Department of Physics, University of Warwick, Gibbet Hill Road, CV4 7AL Coventry, U.K.

## Abstract

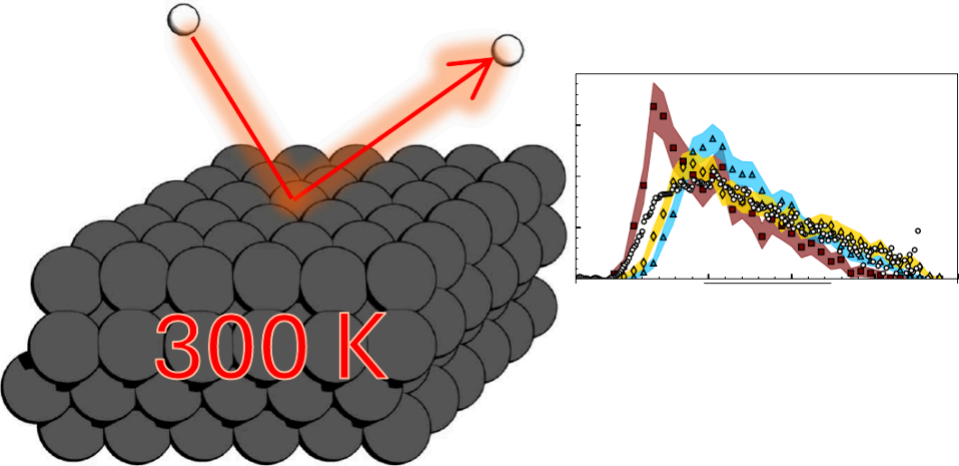

In the dynamics of atoms and molecules at metal surfaces, electron–hole pair excitations can play a crucial role. In the case of hyperthermal hydrogen atom scattering, they lead to nonadiabatic energy loss and highly inelastic scattering. Molecular dynamics with electronic friction simulation results, based on an isotropic homogeneous electron gas approximation, have previously aligned well with measured kinetic energy loss distributions, indicating that this level of theoretical description is sufficient to describe nonadiabatic effects during scattering. In this study, we demonstrate that friction derived from density functional theory linear response calculations can also describe the experimental energy loss distributions, although agreement is slightly worse than for the simpler isotropic homogeneous electron gas approximation. We show that the apparent agreement of the homogeneous electron gas approximation with experiment arises from a fortuitous cancellation of errors as friction is overestimated close to the surface and the spin transition is neglected. Differences in frictional treatment affect single, double, and multibounce scattering trajectories in distinct ways, altering the shape of low-temperature energy loss distributions. These distinctions are largely absent at room temperature but may be measurable in future low-temperature scattering experiments.

The scattering of hyperthermal hydrogen atoms with metal surfaces is a well-studied phenomenon, known to cause substantial energy transfer to the surface.^1^ At room temperature, the energy loss distributions (ELDs) of scattering hydrogen atoms are broad, lacking distinct features with only subtle differences between metal surfaces.^2,3^ Classical molecular dynamics (MD) simulations underestimate this energy transfer because they do not account for nonadiabatic effects during the dynamics and the excitation of electron–hole pairs (EHPs) in the metal.^1,4^ To address this shortcoming, several simulation methods have been developed, among which molecular dynamics with electronic friction (MDEF) stands out as a practically feasible approach. MDEF has been applied to the dynamics of atoms^4,5^ and molecules^6−11^ at metal surfaces, including under the influence of laser pulses.^12,13^ Theoretical studies applying MDEF simulations to H atom scattering^14−16^ were able to primarily link scattering events with low energy loss to single bounce scattering of hydrogen atoms, while events that exhibit high energy losses are associated with atoms that experience multiple bounces.

MDEF simulates the dynamics on the adiabatic ground state potential energy surface while introducing a drag force on the nuclear motion, that accounts for the energy transfer to electron–hole pair excitations.^17^ Remarkably, MDEF simulations have successfully reproduced the ELDs for hydrogen atom scattering from a range of metal surfaces.^2−4^ This success, however, is puzzling for several reasons.

First, in the context of hydrogen atom scattering from a metal surface, the unpaired spin of the hydrogen atom is screened in close vicinity of the metal surface. Upon approaching the surface, the occupied and unoccupied states of the hydrogen atom hybridize with the metal electrons leading to an increase in their spectral width. The spin states become degenerate, and the spin-polarization is lost. This gives rise to a strong nonadiabatic effect that is typically beyond the scope of MDEF.^18^ Indeed, it was previously shown that electronic friction evaluated within time-dependent perturbation theory in the Markov approximation, also referred to as orbital-dependent friction (ODF),^5,8,19^ gives rise to a sudden rise in stopping power at the spin transition geometry.^18,20^ While this effect is ”broadened out” in nonadiabatic mean-field (Ehrenfest) dynamics due to memory effects,^21^ its contribution to the energy loss is similar and significant in both cases.^22^ Previous MDEF studies of hyperthermal hydrogen scattering based on the local density friction approximation (LDFA)^2−4^ have effectively ignored this spin transition. LDFA evaluates electronic friction within a homogeneous electron gas approximation and neglects the electronic structure and spin state of the adsorbate. As a result, previous LDFA studies are expected to underestimate the energy loss in the region of the spin transition, whereas ODF calculations will describe the spin transition in the extreme quasi-adiabatic limit. Both do not fully capture how this effect depends on the details of the trajectory.

Second, while the magnitude of electronic friction from LDFA and ODF align for the homogeneous electron gas,^23^ the validity of LDFA based on DFT electron densities of clean metal surfaces has been previously questioned.^19^

LDFA data presented in studies of hydrogen atoms and molecules chemisorbed at metal surfaces^5,8,19^ overestimate electronic friction and underestimate vibrational lifetimes when compared to ODF. Notably, practical calculations of electronic friction based on ODF within DFT also provide challenges as they require explicit Fermi surface integration that is notoriously difficult to converge. Specific choices regarding mathematical expressions and numerical approximations can affect the results and have previously been discussed in great detail, motivating careful validation against experimentally accessible data.^24^ The hydrogen atom vibrational lifetime on the surface can be measured experimentally and related to the electronic friction coefficient along the corresponding vibrational mode. Reported hydrogen relaxation times by LDFA for Pd(100) (0.2 ps),^25^ Ru(0001) (0.2 ps),^12^ Pb(111) (0.3 ps),^26^ and Pt(111) (0.2 ps, *vide infra*) are a factor of 3–4 faster than experimental measurements for hydrogen atom relaxation on Pt(111) (0.8 ± 0.1 ps)^27^ and Cu(111) (0.7 ps).^28^ As the value of LDFA friction for chemisorbed hydrogen varies only little across close-packed surfaces of transition metals, this may point to an overestimation of friction for chemisorbed hydrogen on transition metals by LDFA. This will likely affect the predicted ELDs of scattered H atoms.

In this work, we simulate hyperthermal hydrogen scattering on Pt(111) for which experiments were previously reported.^2,29^ We perform MDEF simulations with two electronic friction tensor models based on LDFA and full DFT linear response calculations, i.e., ODF.^24,30^ The two models exhibit very different configuration-dependence of friction, yet both predict room temperature ELDs that broadly agree with the experiment. DFT-based electronic friction predicts lower energy loss close to the surface than LDFA, which is in better agreement with time-resolved spectroscopy measurements, but only agrees with the experimental ELD if the spin transition is included. Simulations of low-temperature ELDs, however, reveal clear differences that arise from the specific features of the two friction profiles.

In MDEF, the dynamics are governed by the Langevin equation^17^
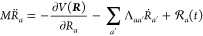
1where *V*(***R***) represents the potential energy surface (PES) which is a function of all atomic positions, ***R***. We calculate elements of the electronic friction tensor, **Λ**, for the three Cartesian coordinates, *a*, of the hydrogen atom projectile.  is the random force, which establishes detailed balance at a given electronic temperature *T* via the second fluctuation–dissipation theorem.^31^ The energy transfer between the translational degrees of freedom of the H atom and the electrons is governed by the second and third terms on the right hand side of eq 1. Within MDEF, the nuclei are treated as classical particles. ELDs from classical simulations of H atom scattering from Xe(111) are in excellent agreement with experiments at ∼40 K which suggests that nuclear quantum effects do not strongly affect ELDs.^100^

We employ a previously reported^32^ high-dimensional effective medium theory^33−35^ (EMT) PES for hydrogen scattering on Pt(111) that was parametrized with PBE-DFT data.^36,37^ The EMT-PES reports a root mean squared error with respect to the DFT data of 259 meV, yet good agreement between the ELDs obtained with this PES and the experiment has been reported.^2^ The shape of the energy loss distributions at room temperature has been shown to be insensitive to the nature and the shape of the underlying PES for H/W(110).^16,38^ We employ a (2 × 2) surface slab model with six metal layers. Electronic friction in the LDFA is calculated from electron scattering phase shifts that arise for a hydrogen ion moving through a homogeneous electron gas with a density equivalent to the density at the position of the hydrogen atom in our simulations.^39^ The resulting friction is a scalar coefficient per atom and only depends on the local electron density, which we evaluate based on the EMT-PES model density.^4,32^

Gaussian process regression^40^ models of ODF friction tensors were generated using scikit-learn(41) (version 1.2.0) with a vector of the inverse hydrogen atom distances to the platinum atoms as a descriptor. ODF tensors without spin polarization were calculated with the electronic structure code FHI-aims^24,42^ (version 210928) for 3198 configurations. The 757 structures with the hydrogen atom above 1.8 Å from the surface were recalculated with system spin constrained to ^2^*S*_1/2_. The MDEF simulations were performed using the NQCDynamics.jl package (development version based on version 0.12).^43^ Further method details are provided in the Supporting Information (SI).

The EMT-PES has been shown to provide a good fit to PBE-DFT data,^29^ which for a hydrogen atom on Pt(111), predicts a binding well with an adsorption minimum in the fcc-hollow site at 0.87 Å (Figure 1a). At large atom-surface separation, the doublet state (^2^S_1/2_) is more stable and the atom exhibits an unpaired spin. At closer distances, the ^2^*S*_1/2_ and ^1^S_0_ states become degenerate in energy as the spin is screened by the metal surface. The adsorption height, *h*, at which this occurs is approximately 2.3 Å, similar to previous findings for H/Cu(111).^20^

**Figure 1:**
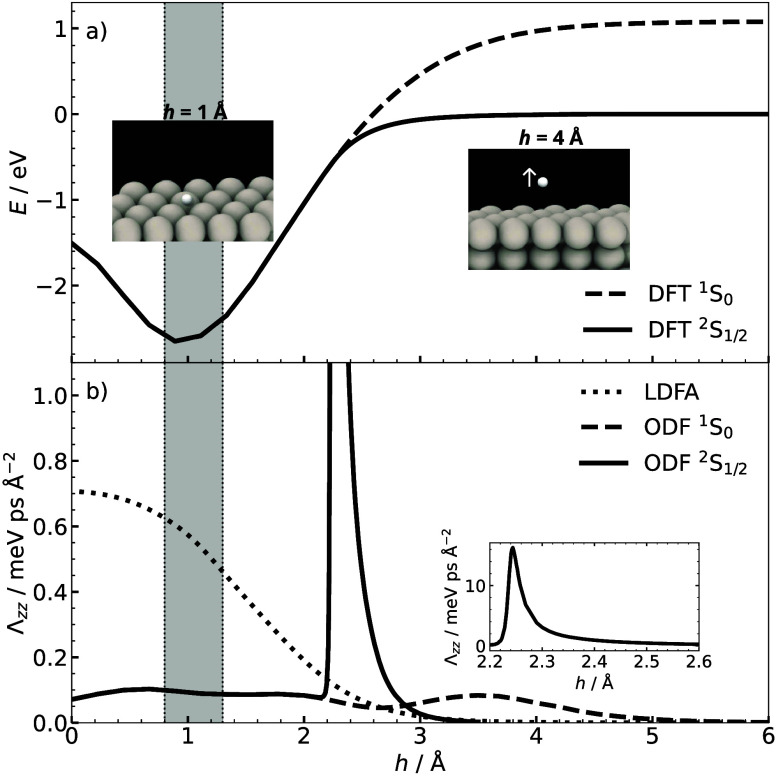
(a) The interaction energy of H on Pt(111) as a function of its atom-surface distance, *h*, above the fcc-hollow site. The solid and dashed lines represent the energy curves for the doublet-constrained spin-polarized and spin-unpolarized DFT calculations, respectively. (b) The friction models as a function of atom-surface distance. The dotted line represents the LDFA values obtained with density values. The dashed and solid lines represent the Λ_*zz*_ values for the constrained spin-polarized and spin-unpolarized DFT calculations, respectively. The inset of panel b shows the entire spin curve for the constrained doublet state with different axis limits. The gray shaded areas in a and b indicate the distances relevant for vibrationally excited, chemisorbed H atoms.

The three friction models differ in their position dependence as a function of atom-surface separation (Figure 1b). As LDFA friction only depends on the electron density of the clean metal substrate, it increases monotonically with decreasing atom-surface separation from a height of about 3 Å. It reaches a value of around 0.5 meV ps Å^–2^ at around 1 Å. LDFA does not account for changes in the magnetization density of the projectile.^44^ The coefficient of the ODF friction tensor perpendicular to the surface (Λ_*zz*_) is sensitive to the spin state of the system as the atom approaches the surface. In the ^2^S_1/2_ state, ODF friction starts to rise at the same atom-surface distance as LDFA, but exhibits a sharp rise to approximately 15.6 meV ps Å^–2^ at the separation distance of 2.3 Å above the fcc-hollow site, where the spin is screened by the metal and doublet and singlet states become degenerate. Below this height, the friction values are identical for both spin states. The ODF ^1^S_0_ extends much further into the vacuum, away from the surface, which is an artifact that arises from enforcing an incorrect spin state at these geometries.

The gray shaded region in Figure 1 represents the height ranges relevant for chemisorbed H atoms. In this region, there is approximately a factor 5 difference in the friction predicted by LDFA and ODF. The lifetime of the vibrational stretch mode of a chemisorbed H atom at the surface including EHP excitations and lattice vibrations is determined through MDEF simulations for H on Pt(111) adsorbed at the energetically favorable top site using the EMT-PES potential and the respective friction models. A detailed procedure of the simulations is given in the SI and the results are reported in Table 1.

**Table 1:** Comparison of Experimental and Computed Vibrational Lifetimes of the Coherent H–Pt Stretch Mode of Hydrogen at the Top Site^a^

τ_***q***=0_^LDFA^ (ps)	τ_***q***=0_^ODF^ (ps)	τ^exp^ (ps)
0.174 ± 0.01	1.15 ± 0.2	0.8^27^

a The theoretical values were acquired with MDEF simulations using LDFA and the ^2^S_1/2_ ODF model.

The vibrational lifetime predicted by MDEF based on LDFA is lower than the result based on ODF and the experimentally measured value. Note that electronic friction in the ^1^S_0_ and ^2^*S*_1/2_ states is identical close to the surface and it is, therefore, sufficient to report only one of the two. ODF-based MDEF simulations yield a lifetime that is slightly larger than the experimental value. We expect the prediction to be an upper bound to the experiment as we neglect higher-order phonon–phonon coupling. LDFA overestimates the dissipation rate due to electron–hole pair excitations in the chemisorption region, whereas the friction values from ODF are more consistent with experiment.

Based on the three different friction models, we simulate hyperthermal ELDs at a kinetic incidence energy of 1.92 eV and a substrate temperature of 300 K. (Figure 2) Our LDFA-based MDEF results are consistent with the simulations reported by Dorenkamp et al.^2^ and Lecroart et al.,^29^ all of which agree well with experiment. The LDFA ELD predicts a lower likelihood of low energy loss scattering than the experiment, but the peak position and tail of the distribution are captured well. MDEF simulations, performed with the ^1^S_0_ ODF model, provide a distribution that is too steep at low energy losses and predicts the most likely energy loss to be around 0.2 eV significantly below the experimental peak at around 0.5 eV. Furthermore, scattering events with energy losses beyond 1 eV are underestimated by this model. Hence, ODF-based MDEF results without inclusion of the spin transition are not consistent with experimental findings. MDEF simulations with the ^2^S_1/2_ ODF model predict an energy loss distribution that manages to qualitatively reproduce the experimental ELD too, but on a quantitative level, LDFA does better. The ^2^S_1/2_ ODF curve is able to capture the most probable energy loss around 0.5 eV seen in the experiment, and also captures the decaying tail at high energy losses correctly within the statistical uncertainties.

**Figure 2:**
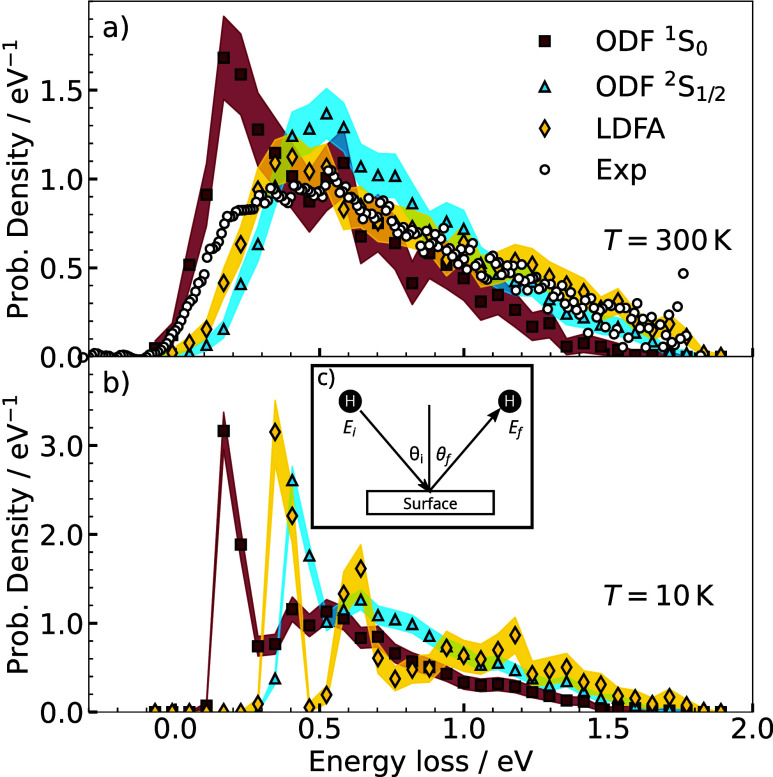
(a) Computed energy loss distribution obtained from MDEF simulations of specular scattered H atoms from Pt(111) with different friction models. The experimental data is taken from ref (2). The H atoms were launched along the [101̅] direction with an initial kinetic energy *E*_kin,i_ of 1.92 eV and an incidence angle of θ_i_ = 45°. The surface temperature was set to 300 K. The colored, transparent schemes indicate the 95% confidence interval of the calculated ELDs. (b) Energy loss distributions simulated at a surface temperature of 10 K. All other parameters are the same as in panel a. (c) Inset depicts the scattering geometry of the simulations.

The ELDs predicted by LDFA and ODF ^2^S_1/2_ at room temperature are remarkably similar despite their significantly different friction profiles (Figure 1b). The random force term in MDEF causes a broadening effect in the predicted room temperature ELDs^38^ which will mask the underlying differences in the description between these two models. For example, previous theoretical works^14,15^ have shown the room temperature ELDs predicted by MDEF can be decomposed into strongly overlapping signals from single, double and multibounce events. As such, single scattering events at 300 K cannot be separated clearly from multibounce events. We find that the fraction of adsorbed particles depends on the employed friction model, with LDFA yielding larger sticking coefficients than either ODF model and the ^2^S_1/2_ ODF model predicting higher sticking coefficients than the ^1^S_0_ model (Figure S11). Unfortunately, we are unaware of any reported experimental sticking coefficients for monoenergetic, hyperthermal H atoms impinging on Pt(111) but we propose that such experiments could provide additional support in validating different nonadiabatic simulation techniques.

Furthermore, as previously shown, at low temperatures, energy loss distributions from hydrogen scattering are much more finely resolved and the energy region of single and multiscattering events can be better distinguished.^38^ ELDs from MDEF simulations at 10 K (Figure 2b) reveal that the differences in the electron friction profiles have a far greater effect at low temperatures. The two ODF models generate similarly shaped ELDs characterized by a distinguishable, sharp first peak and a decaying broad tail, though with a shift to higher energy losses for the ^2^S_1/2_ model. It is evident that the electronic friction peak caused by the spin transition introduces a rigid shift between the two ELDs. In contrast, the LDFA model exhibits a clearly separated sharp first peak and a sharp second peak, followed by a broad tail.

We examine the relationship between the friction profiles predicted by each model and the low-temperature ELDs by scaling parts of the friction profiles and studying the effects on the simulated ELDs. Reducing LDFA by a factor of 3.5 provides an ELD (Figure 3a) that closely aligns with that predicted by ODF ^1^S_0_, where the previously distinct second peak and decaying tail have now merged. The rescaled LDFA roughly matches with the average of the diagonal elements (Λ_iso_) of ODF close to the surface (see insets of Figure 3). Similarly, increasing ODF ^2^S_1/2_ close to the surface (below the spin transition peak) by the same factor produces a distribution with distinct first and second peaks, akin to LDFA, except with a shift to higher energy losses from the presence of the spin transition peak (Figure 3b). These findings show that by increasing the friction close to the surface, we increase the energetic separation between the peaks in the ELDs at low temperature. Previous analysis^14,38^ connects the distinct peaks in the low-temperature ELD to the number of bounces the H atom undergoes in the trajectory (first peak is due to single bounces, the second peak is due to double bounces, etc.). Thus, by increasing the friction at the surface, there is an increasing translational energy loss that is incurred with each bounce from the surface. This principle is shown for an example trajectory in Supplementary Figure S4, where each bounce accrues the greatest energy loss with the LDFA model.

**Figure 3:**
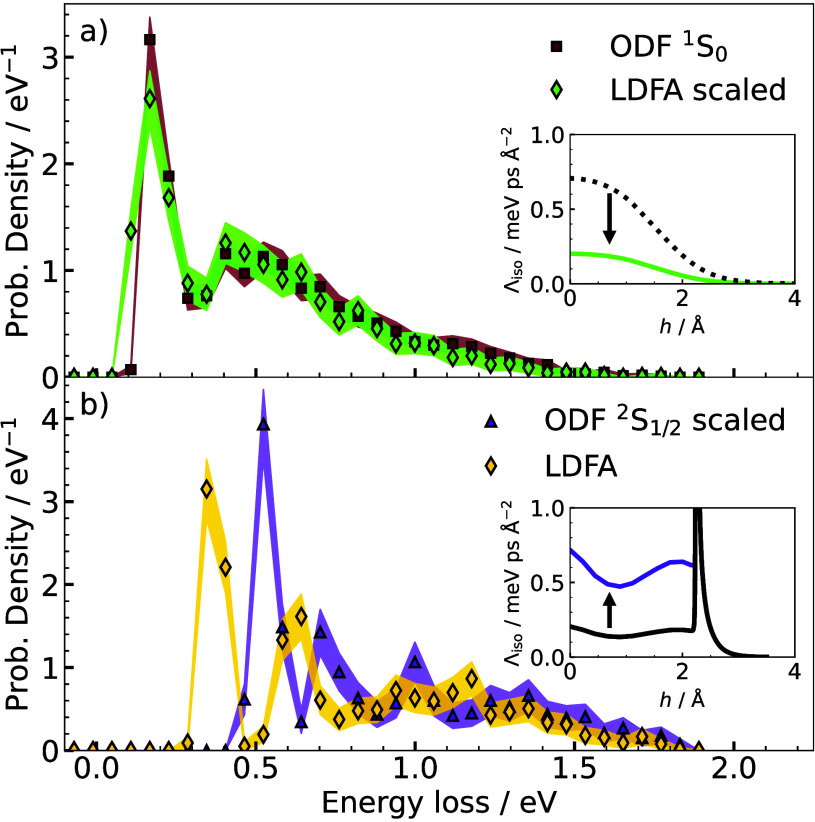
Comparison of specular energy loss distribution obtained from MDEF simulations with different friction models at a surface temperature of 10 K. (a) Simulations with a scaled-down LDFA model to match the ^1^S_0_-ODF-based ELD from Figure 2. The inset replots Figure 1b for the scaled-down LDFA model (green, solid line) and the original LDFA model (black, dotted line). (b) The ELD from MDEF simulations with a scaled-up ^2^S_1/2_ model and with the original LDFA model. The inset plots isotropic friction values (i.e. the average of diagonal elements, Λ_iso_) for the scaled (purple) and original (black) ^2^S_1/2_ ODF-model.

This study highlights the strengths and limitations of LDFA and ODF for describing nonadiabatic energy loss in high-dimensional hydrogen atom scattering simulations. The LDFA overestimates friction close to the surface compared to electronic friction calculated from first-order response theory based on density functional theory, so-called ODF, and experimental measurements of the vibrational lifetime of the H atom stretch on Pt(111). LDFA furthermore neglects the spin transition that occurs during hydrogen atom impingement. Despite both shortcomings of LDFA, our simulations show that the prevalent room temperature hydrogen atom scattering experiments are well described by simulations with LDFA, closely followed by simulations with ODF where the spin transition is considered in the quasi-adiabatic limit. Therefore, the experiments are not sensitive to even stark and qualitative differences that arise between the two electronic friction profiles. The discrepancy between LDFA and ODF has been previously discussed in literature. Previous work on reactive scattering of N_2_ on Ru(0001) has shown that ODF and LDFA yield different results, where ODF was found to provide a better description of the experiment.^9^ The case of H atom scattering is further complicated by the presence of a spin transition that is neither correctly described by LDFA nor ODF and that likely limits the applicability of electronic friction theory.

The specular scattering that is measured corresponds to a highly integrated signal. At room temperature, dynamic fluctuations broaden signatures of single, double and multibounce scattering events, which merge into a single broad distribution. The exact profile of friction determines the energy loss incurred during the initial approach to the surface and the subsequent dynamics at the surface. If friction is large in close vicinity to the surface, single and double bounce events are expected to be energetically well separated in the measured ELDs. If experimentally resolved, this would provide an indirect measurement of the magnitude of electronic friction that would be complementary to vibrational lifetime measurements. At low temperature, details of the energy landscape and the exact profile of friction will become much more apparent in the energy loss distribution. We suggest that low-temperature experiments are conducted in the future, which will support the development of more accurate energy landscapes and help to distinguish signatures of electronic friction related to the chemisorption region and the spin transition during hyperthermal hydrogen scattering. Such experiments will provide deeper insights into the mechanisms of nonadiabatic energy loss during chemical dynamics at metal surfaces and constitute reference results that will further advance the theoretical description of nonadiabatic dynamics at surfaces.

## Supporting Information Available

Computational details of DFT calculations, simulations, and machine learning models, as well as additional simulation data (PDF)
