# Supplementary material for: Room Temperature Hydrogen Atom Scattering Experiments Are Not a Sufficient Benchmark to Validate Electronic Friction Theory
Source: J Phys Chem Lett. 2024 Dec 13;15(51):12520–5. doi: 10.1021/acs.jpclett.4c02468 (PMC11684155; doi:10.1021/acs.jpclett.4c02468)
Supplement: Supplementary file 1 — jz4c02468_si_001.pdf [file jz4c02468_si_001.pdf]

Room Temperature Hydrogen Atom Scattering Experiments Are Not a Sufficient Benchmark to Assess Electronic Friction Theory

Connor L. Box,† Nils Hertl,† Wojciech G. Stark,† and Reinhard J. Maurer*,†,‡

†*Department of Chemistry, University of Warwick, Gibbet Hill Road, CV4 7AL, Coventry, UK.*

‡*Department of Physics, University of Warwick, Gibbet Hill Road, CV4 7AL, Coventry, UK.*

E-mail: r.maurer@warwick.ac.uk

Contents

1 DFT calculations **2**

2 Simulation details **3**

3 Friction models **5**

4 Energy loss profile **8**

5 The role of non-diagonal elements of EFT **12**

6 Calculation details of dynamical lifetimes **14**

7 Sticking coefficients **18**

References **19**

1 DFT calculations

Our DFT calculations were performed with the FHI-aims code (2021 development version).[1] To acquire the necessary input data for the ODF model from DFT, we used a 2×2 cell with 6 layers with a single H atom in the cell to model atomic hydrogen interacting with a Pt(111) surface. The two bottom layers were kept frozen in their bulk truncated positions. Periodic boundary conditions were employed in all three directions. The cell height (perpendicular to the slab) was 70 Å, to prevent the interaction of the slab with its periodic images in the vertical direction. The lattice constant was taken from Ref. 2. For the relaxation of the slab, we used a standard 'tight' basis set. The reciprocal space was sampled with a $16 \times 16 \times 1$ $\boldsymbol{k}$-point mesh according to the sampling method proposed by Monkhorst and Pack,[3] and an atomic ZORA relativistic correction.[4] A Gaussian width of 0.1 eV was used for the occupation smearing in the self-consistent field cycles. The PBE exchange-correlation functional[5,6] is employed with 10^{-6} eV, 10^{-3} eV and 10^{-5} e/a_0^3 tolerances for the total energy, the eigenvalue energies and the electronic density, respectively. The convergence criterion for the forces was set to 10^{-4} eV/Å.

The ODF tensors were calculated using FHI-aims,[1] and we refer to Ref. 7 for a detailed description of the underlying formulae of ODF implemented in that electron structure code. The same computational settings were employed as above, except a standard FHI-aims 'light' numerical basis set was used. To assess the effect of the spin on the friction tensor, we conducted calculations with and without spin polarisation and kept the total spin of the system fixed. We used a Gaussian width, σ, of 0.6 eV to replace the delta distribution in the expression of the ODF friction tensor. The ODF calculations are treated at an electronic temperature of 0 K.

The spin-polarised ODF coefficients are not significantly different between $\sigma = 0.1$ eV and $\sigma = 0.6$ eV for a range of heights above the hcp site (as shown in Figure S1) except in the range of heights $1-2$ and at the maximum of the spin-transition peak. These differences are not expected

to strongly affect the energy loss distributions nor affect the conclusions drawn in the main paper.

We also assessed the convergence with respect to the lateral cell size and found that the diagonal elements are converged in a $p(2 \times 2)$ cell as shown in Figure S2.

2 Simulation details

The MDEF simulations were carried out using the NQCDynamics.jl[8] package (development version based on version 0.12) that interfaces with the md_tian2 code,[9] which provides the EMT-PES for the energies, and forces required for the propagation of the atoms. The parameters that define the H/Pt(111) EMT-PES have been taken from Ref. 10. The BAOAB Langevin propagator proposed by Leimkuhler *et al.*[11] has been used for all atoms in the simulation cell except that we only provide non-zero friction elements for the H atom to account for ehp excitation during the scattering process. Slab atom positions were randomly sampled with thermal Monte Carlo simulations, which were resampled for every 100 scattering trajectories. Velocities for the slab atoms were obtained from a Boltzmann distribution. Incidence kinetic energy, incidence angle and crystallographic incidence direction were given as initial conditions, which define the initial entries of the H atom's velocity vector. The initial lateral positions of the H atom were chosen randomly and the projectile was positioned 6 Å above the surface. The simulation time and the time step were chosen manually for the chosen friction method and initial kinetic energy. Table S1 gives an overview of the simulation details. To meet the experimental conditions as close as possible, we only used

Table S1: Chosen MDEF simulation parameters.

Model	$E_{\mathrm{kin,i}}$	Time step / fs	Simul. time limit / fs
LDFA	1.92	0.1	300
ODF $^1S_0$	1.92	0.1	300
ODF $^2S_{1/2}$	1.92	0.05	300

those scattering events for the calculation of the energy loss distribution, which scattered in-plane with a tolerance angle of 6° at a scattering angle of 45±5°. The shaded regions of Figure 2 in the

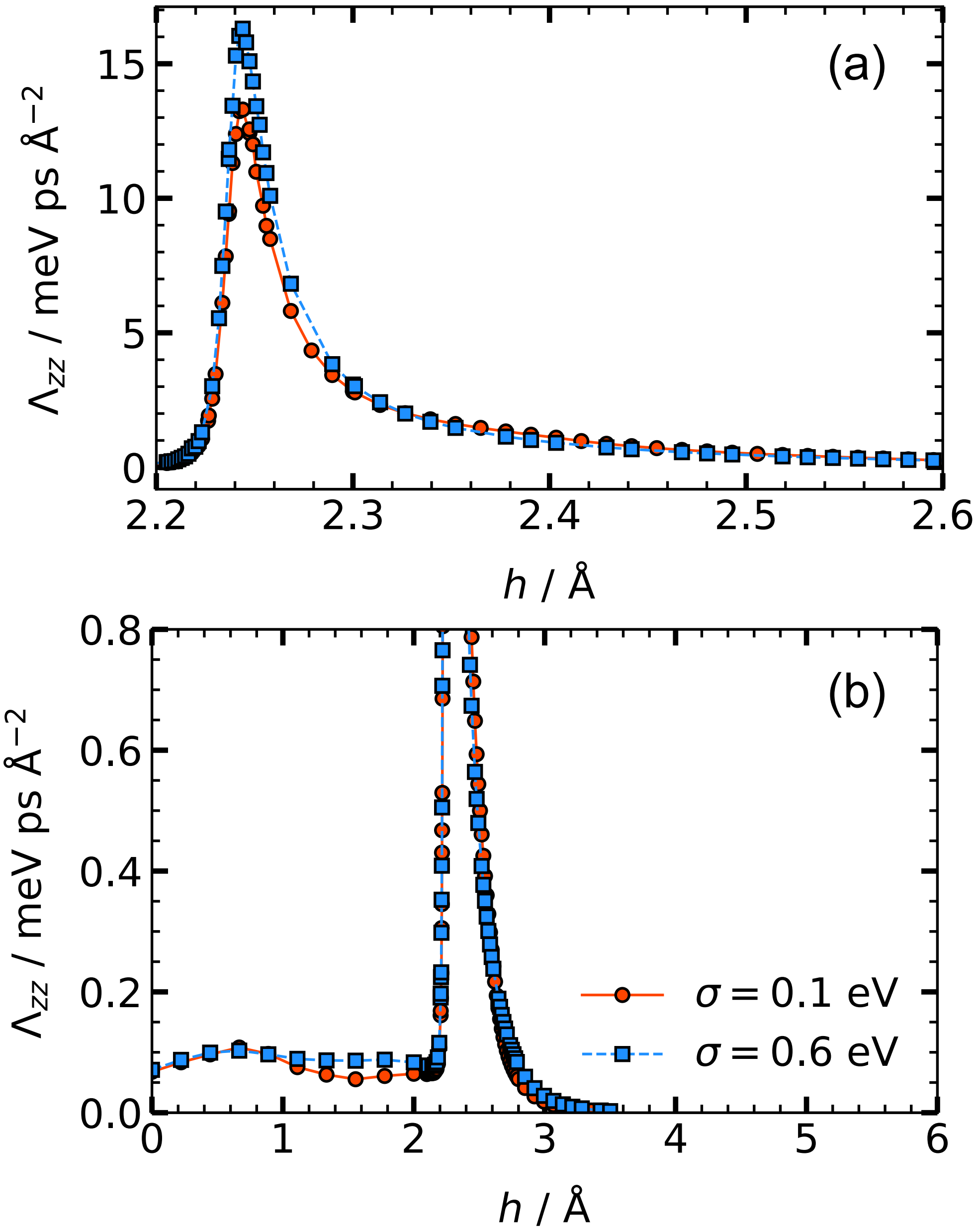

Figure S1: Same as Figure 1(b) in the main manuscript, except only $^{2}S_{1/2}$ ODF is shown for two different broadening parameters, σ. The two subplots are shown for different axis limits.

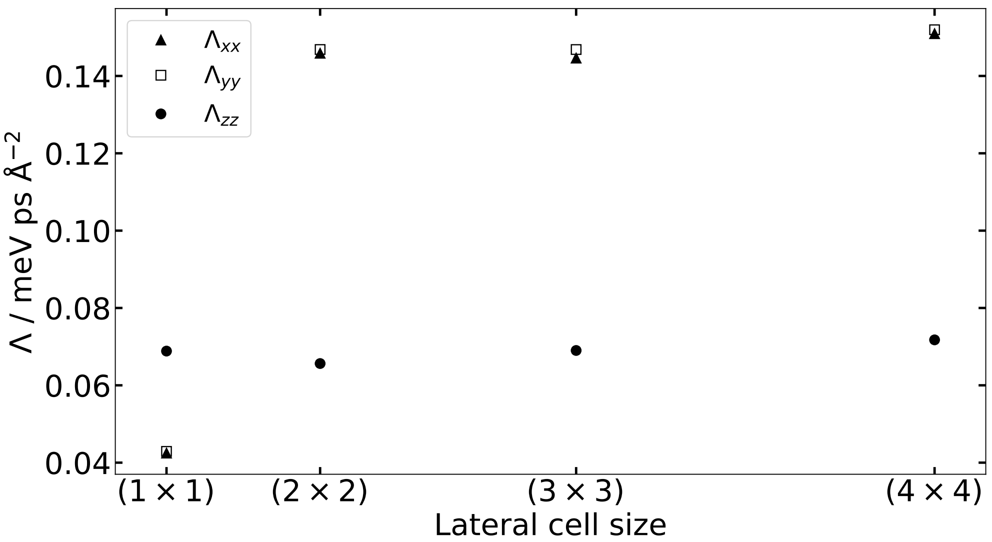

Figure S2: Diagonal elements of the ODF friction tensors as a function of the lateral cell size for a H atom in its minimum energy configuration at the fcc-hollow site, depicted in Figure 1a).

main manuscript give the 95 % confidence intervals, C which are calculated by the following:

$$C = \frac{1.96}{w} \times \sqrt{\frac{p(1-p)}{N}}, \tag{1}$$

where w is the bin width of the ELDs, p is the probability density and N is the number of trajectories.

3 Friction models

Within the LDFA framework, the friction tensor is isotropic and characteriby a single scalar, $\mathbf{\Lambda} = \Lambda \mathbb{1}$, where Λ is determined by the electron density. A notable advantage of the EMT approach is that the total energy expression depends on a model density, n_{EMT}, which can be used to compute the friction coefficients at the LDFA level.[12,13] We employed the mapping function between model density and friction coefficient provided in md_tian2, as detailed in Ref. 12.

When ODF coefficients were employed, they came from one of two Gaussian process regression[14] models: one without spin polarisation and one with spin polarisation combined with a 1D fit

for H atom heights above the beginning of the spin transition. The GPR[15] models were generated using `scikit-learn`[16] (version 1.2.0) with a vector of the inverse hydrogen atom distances to the platinum atoms used as a descriptor.

3198 data points were collected via spin-unpolarised DFT calculations, and the 757 structures with the hydrogen atom above 1.8 Å from the surface were recalculated with spin-poloarisation to form the two datasets. Of the data points, 95 % were chosen to be training data, the remaining 5 % formed the test data. As a consequence of the exclusion of configurations where the hydrogen atom sits below the second subsurface platinum layer in the training data, we do not employ the generated ODF models for incidence energies where significant subsurface scattering is expected. The `scikit-learn` convenience function, `MinMaxScaler`, is employed on the training and test data to scale the features to a range between 0 and 1.

Only the diagonal elements of the electronic friction tensor (EFT) were included in the GPR models and the subsequent MDEF simulations. The $^1S_0$-ODF model achieved a RMSE of 0.0076 ps^{-1} for the diagonal elements of the EFT for 5 unseen validation trajectories, an example of which is shown in Figure S3 The GPR models quantitatively predict the $^1S_0$-ODF diagonal elements across this example trajectory, with the accuracy highest and GPR standard deviation lowest for the z diagonal element.

Direct fitting of the $^2S_{1/2}$-ODF coefficients using the same methodology was not possible, due to the presence of the large peak in diagonal element associated with motion in z coordinate (Λ_{zz}), as a consequence of the screening of the projectile spin, as discussed in the main manuscript. Instead, a GPR model was generated for the ODF coefficients below 2.21 Å and the mass-weighted friction ($\tilde{\Lambda}$) in the spin-transition region was fitted separately using:

$$\tilde{\Lambda}_{zz}(h) = \begin{cases} p(h, h_a, h_0, h_\omega, h_\sigma), & 2.21\text{ Å} \leq h \leq 2.24\text{ Å} \\ p(h, h'_a, h'_0, h'_\omega, h'_\sigma), & 2.24\text{ Å} < h \leq 2.5\text{ Å} \\ g(h, h''_a, h''_t, h''_\omega, h''_\sigma), & h > 2.5\text{ Å} \end{cases}, \tag{2}$$

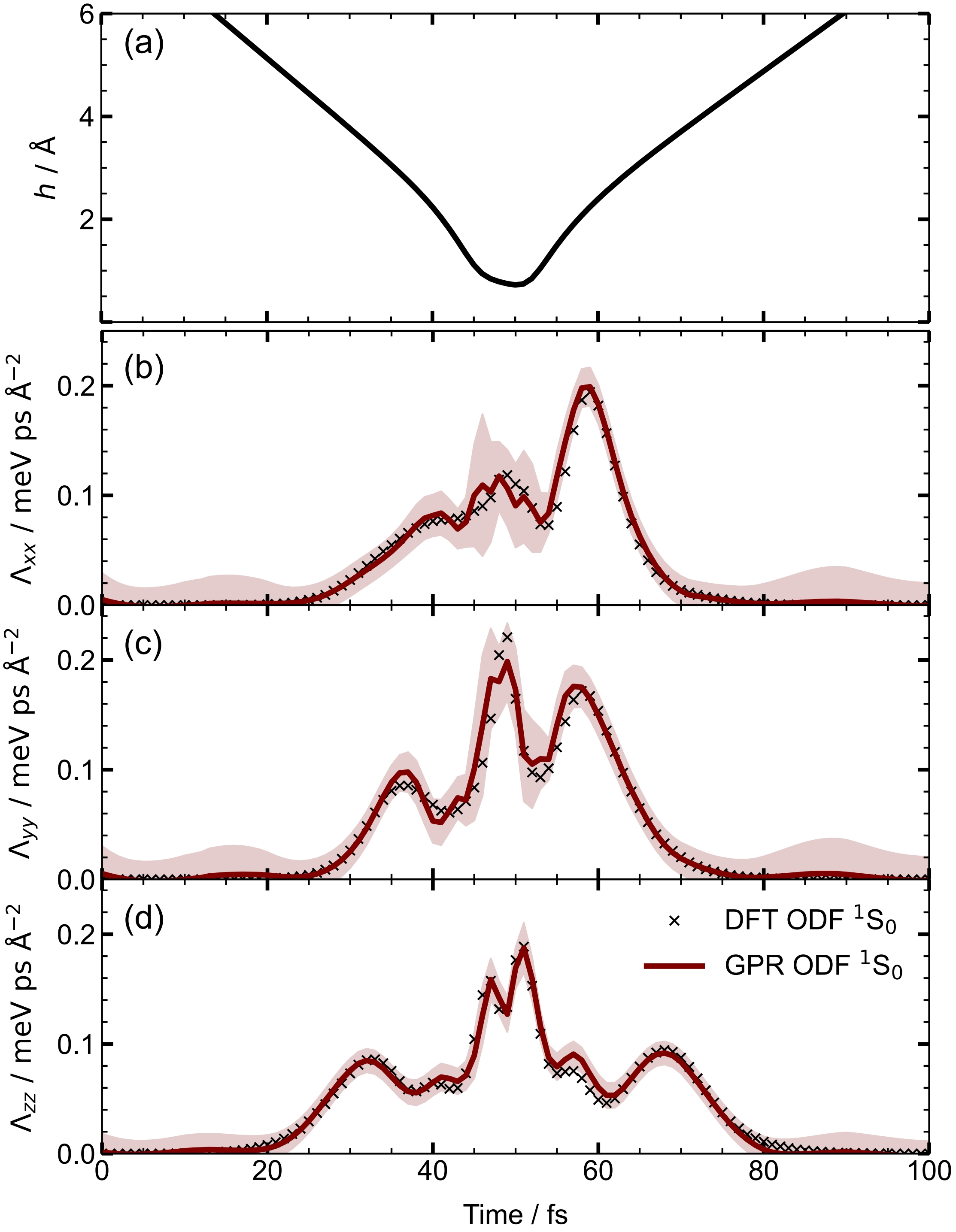

Figure S3: For an example trajectory ($E_\mathrm{i} = 1.92$ eV) the (a) height of the hydrogen atom projectile and (b-d) diagonal ODF $^1S_0$ friction tensor elements calculated with spin unpolarised DFT and the GPR models are shown as a function of simulation time. Trajectory is unseen in the training of the GPR models. Shaded region is the standard deviation provided by the GPR model.

where the Pearson type VII-like distribution,[17] p was used as a functional form:

$$p(h,h_a,h_0,h_\omega,h_\sigma) = \frac{h_a}{\left[1+\left[h_\sigma^{-1}\left(2^{1/h_\omega}-1\right)^{\frac{1}{2}}(2h-2h_0)\right]^2\right]^{h_\omega}}, \tag{3}$$

and the exponential decay (g) function is given by:

$$g(h,h_a,h_0,h_\omega,h_\sigma) = h_a * \exp(-h_0 * h + h_\sigma) + h_\omega. \tag{4}$$

The employed parameters are given in Table S2. The fitted curve described above is used for the Λ_{zz}

Table S2: Parameters employed for the curve fitting of the spin-peak in the $^2S_{1/2}$-ODF coefficients as employed in Equation 2

h_a	1.0005 ps^{-1}	h'_a	1.0546 ps^{-1}	h''_a	13.1754 ps^{-1}
h_0	2.2430 Å	h'_0	2.2397 Å	h''_0	6.0807 Å^{-1}
h_ω	2.8186	h'_ω	0.6428	h''_ω	0.0000 ps^{-1}
h_σ	0.0175 Å	h'_σ	0.0452 Å	h''_σ	78.9657

coefficients when the hydrogen atom is above 2.21 Å whilst the GPR model is used everywhere else. The performance of this model for Λ_{zz}, shown for an example trajectory in Figure S4, is adequate to qualitatively explore how a peak in friction in the spin transition region affects the ELDs (see the main manuscript).

4 Energy loss profile

In this section, we provide details on how the energy loss profile along the trajectory in Figure S5 was acquired. At first, a Born-Oppenheimer molecular dynamics (BOMD) trajectory was calculated without non-adiabatic effects but all other conditions are the same as those used in the simulations for the ELDs shown in Figure 2. The H atom's position was used to compute the friction tensor which the projectile would face in a MDEF trajectory at that location. The extracted friction tensor and velocities were subsequently used to calculate the energy transfer due to electronic

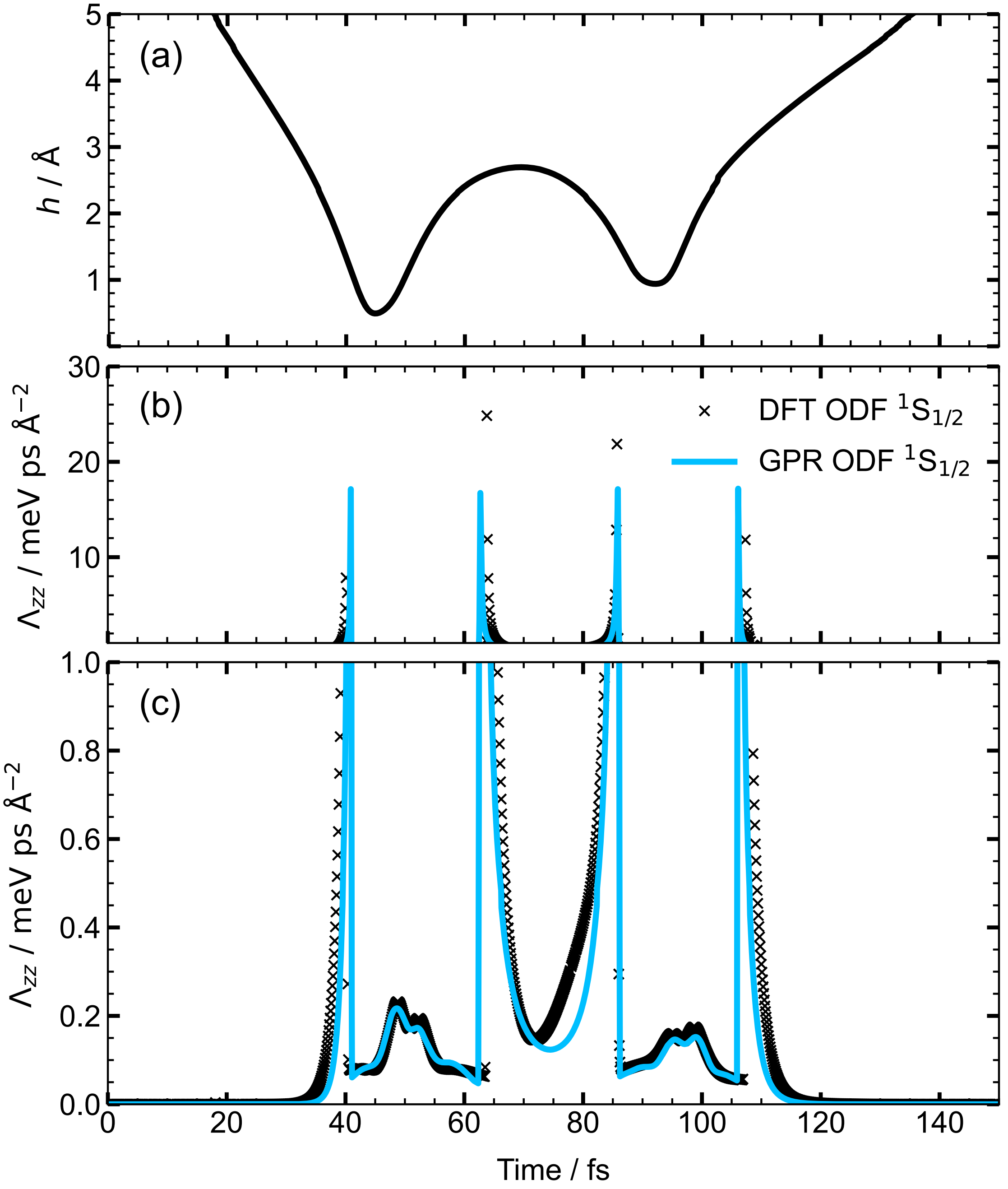

Figure S4: For an example trajectory ($E_{\mathrm{i}} = 1.92$ eV), the (a) height of the hydrogen atom projectile and (b-c) ODF $^2\mathrm{S}_{1/2}$ Λ_{zz} element calculated with spin-polarised DFT and GPR model are shown as a function of simulation time. Trajectory is unseen in the training of the GPR model. Different y-axis limits are shown in (b) and (c) for visual clarity.

friction along the trajectory with the following equation:

$$E_{\mathrm{ehp}} = \int_0^t \mathrm{d}t' \dot{\vec{r}} \Lambda(\vec{r}) \dot{\vec{r}} \approx \sum_{i=1}^{N} \dot{\vec{r}}_i \Lambda(\vec{r}_i) \dot{\vec{r}}_i \Delta t, \tag{5}$$

where $\mathbf{\Lambda}(r)$ and $\dot{\vec{r}}$ are the position-dependent friction tensor and the projectile's velocity, respectively. The index i marks the ith integration step and N stands for the trajectory's total number of integration steps. The velocities taken from the BOMD trajectory were not affected by the frictional drag during the run and are thus higher compared to an actual MDEF trajectory. Hence, for each degree of freedom, we subtract the accumulated energy loss along that respective degree of freedom from the magnitude of the adiabatic velocity component $|\dot{r}_{\alpha,j}^{\mathrm{BOMD}}|$, i.e.,

$$|\dot{r}_{\alpha,j}| = |\dot{r}_{\alpha,j}^{\mathrm{BOMD}}| - \sqrt{2\frac{\sum_{i=1}^{j-1} \Lambda_{\alpha\alpha}(\vec{r}_i) \dot{r}_{\alpha,i}^2 \Delta t}{m_{\mathrm{H}}}}. \tag{6}$$

Here α labels the Cartesian elements and j stands for the jth integration step of the BOMD trajectory. The sum in the second term of Eq. 6 runs over all previous integration steps.

For the example trajectory in Figure S5 a) one can infer from the H atom height that there are two bounces on the Pt(111) surface, the first time around 50 fs and the second time around 200 fs. Hence, along this trajectory, the particle undergoes a spin transition four times in the Λ_{zz} profile of the $^2S_{1/2}$-ODF model, shown in Figure S5b) where the friction reaches its maximum values. In contrast the maxima of the LDFA can be found when the H atom is closest to the Pt surface. The $^1S_0$-ODF model provides significant friction at much larger distances from the surface, which is a consequence of the H atom's wrong spin state at those distances. These differences have an effect on the time profile of the energy dissipation in Figure S5 c). In the case of LDFA, steep increases in energy dissipation are localised to the two bounces with the surface. The $^1S_0$-ODF model exhibits lower energy dissipation than LDFA at the bounces, but continually dissipates energy far from the surface (in the time spent between bounces). In the case of the $^2S_{1/2}$-ODF model, the energy dissipation exhibits steep jumps when the H atom passes through the spin transition region, with

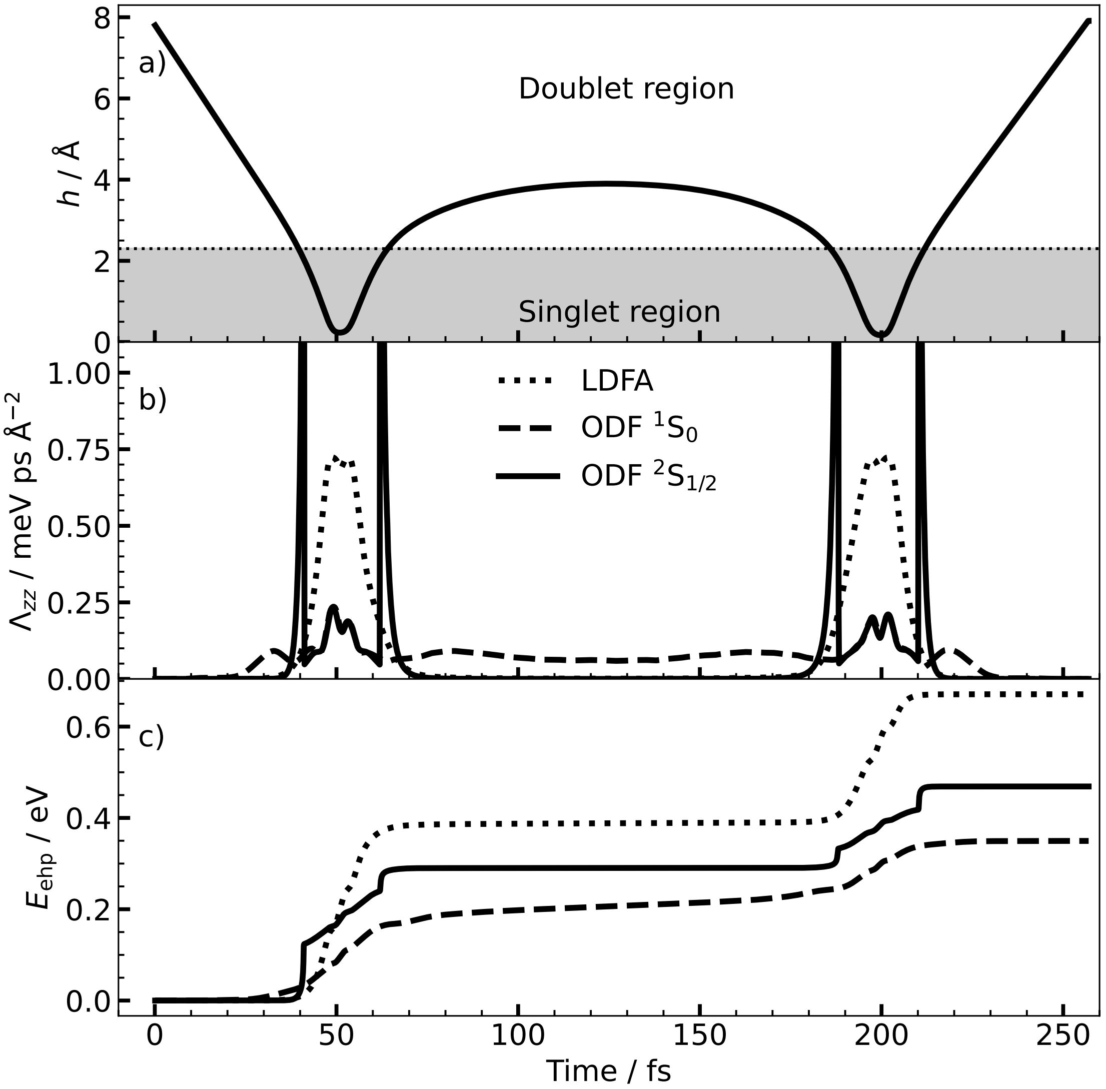

Figure S5: Altitude, friction and non-adiabatic energy loss profile acquired from the positions and velocities of an adiabatic trajectory with two collision events. Panel a) depicts the altitude of the projectile, where $h = 0$ Å corresponds to the surface layer. The grey-shaded area corresponds to the region where the H atom's spin is screened by the metal electrons—see Figure 1a) for details. Panel b) shows the friction profile for the three friction models along the time evolution of the scattering trajectory. Panel c) shows the corresponding accumulated energy loss over time.

a lower rate of energy dissipation close to the surface than LDFA. Note that the first pass through the spin transition regions makes the largest contribution of all four passes, where the velocity is the highest. In this example trajectory, the difference in energy loss between the first and second bounce is greatest for LDFA, and similar for both ODF models. This is expected to account for the distinction in the peaks associated with single bounce and double bounce trajectories in the low-temperatue ELDs for LDFA compared to the ODF models.

5 The role of non-diagonal elements of EFT

The off-diagonal ODF elements were excluded from the GPR models used for the results in the main manuscript. The off-diagonal elements have a small magnitude except for the closest distances between the projectile, shown for an example trajectory in Figure S6. At these distances, the hydrogen atom velocity is small, and the subsequent friction-induced energy loss is expected to be negligible. The off-diagonal elements are thus not expected to contribute significantly to the energy loss distributions.

To assess the impact of off-diagonal elements of EFT on the scattering dynamics, we constructed a model based on a method by Sachs *et al.*[18] This approach utilizes the atomic cluster expansion (ACE)[19] methodology to construct full EFTs. The model was trained using Adam optimizer, with a learning rate of 10^{-4}, and exponential decay rate for the momentum and velocity terms of 0.99, and 0.999, respectively. A column-wise coupling was used in constructing a representation of atoms together with a cutoff distance of 5 Å, a maximum correlation order of 2, and a maximum polynomial degree of 12. Scalar- and matrix-equivariant sub-blocks of the diffusion coefficient matrix were included. The model RMSE evaluated on a test set consisting of 634 random H/Pt structures was 0.092 ps^{-1} (Fig. S7).

In Fig. S8, the energy loss is shown for the same scattering trajectory as in Fig. S5 c. Here, the energy loss obtained using the GPR ODF model is compared with the energy loss profile generated using the ACE model in two versions. In the first version, only diagonal elements were

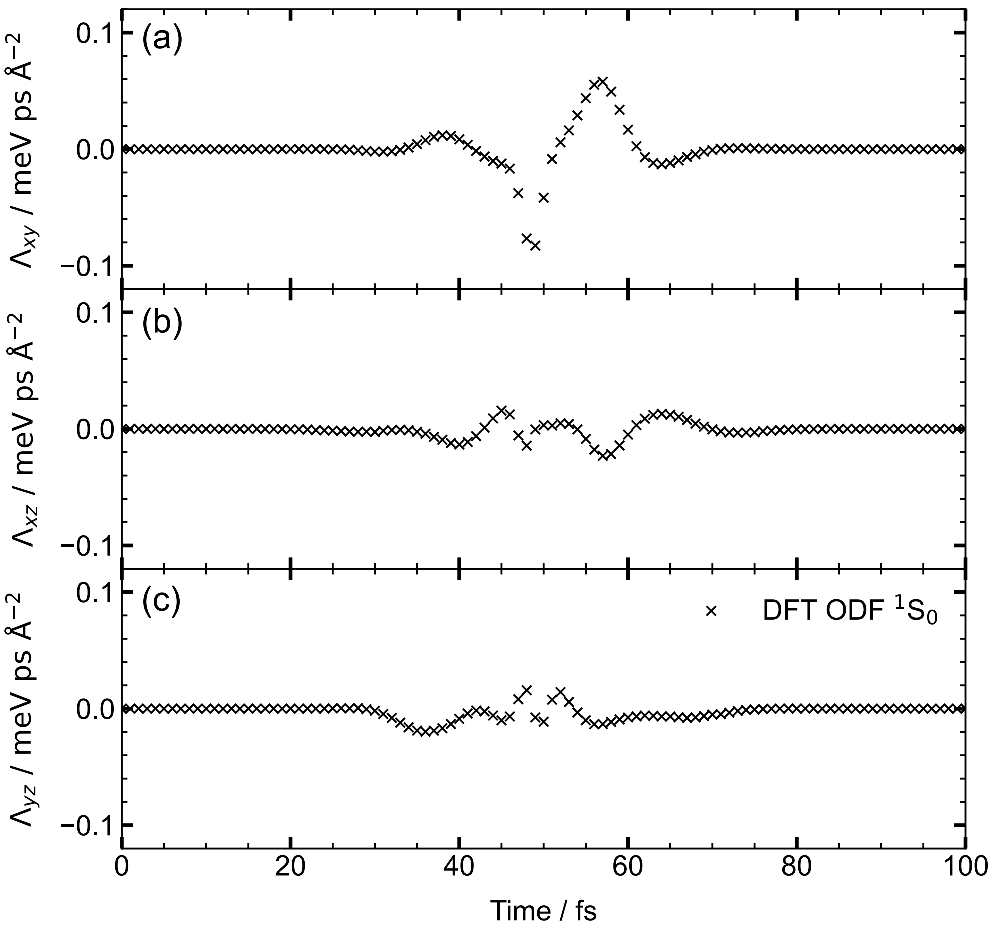

Figure S6: For an example trajectory ($E_\mathrm{i} = 1.92$ eV) the (a-c) off-diagonal ODF $^1\mathrm{S}_0$ friction tensor elements.

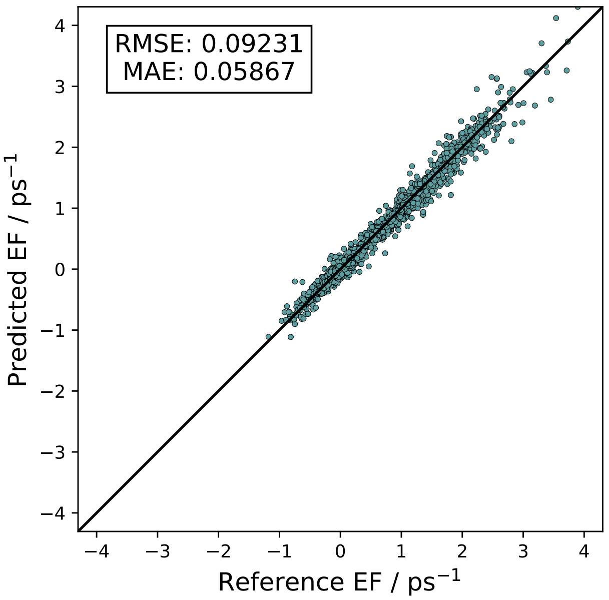

Figure S7: Performance of ACE-based EFT model based on ODF. Predictions of mass-weighted electronic friction tensor elements in ps^{-1} made using an ACE-based model with corresponding DFT-based reference results. All diagonal and off-diagonal elements are compared.

used, whereas in the second version, all the elements of EFT were included. The energy dissipation generated with the ACE model follows the curve generated with the GPR model very closely. Similarly, the agreement between the diagonal-only and full tensorial ACE models is almost perfect. Nondiagonal EFT elements are roughly 10 times lower than the diagonal elements, throughout an entire scattering trajectory (Fig. S9). This suggests that the impact of nondiagonal elements of EFT on the scattering dynamics of H at Pt(111) surface is minor and thus neglecting the nondiagonal elements is justified.

6 Calculation details of dynamical lifetimes

In order to obtain vibrational lifetimes that include anharmonic effects, we employed MDEF simulations where the H atom in an adsorption geometry at a top site is initiated with kinetic energy equal to the experimental[20] vibrational energy for $v = 1$ (0.259 eV). These simulations follow the same procedure to initiate the metal atoms as the scattering simulations described above. Subsequently, we let the system propagate with a time-step $\Delta t = 0.1$ fs and monitored the kinetic energy

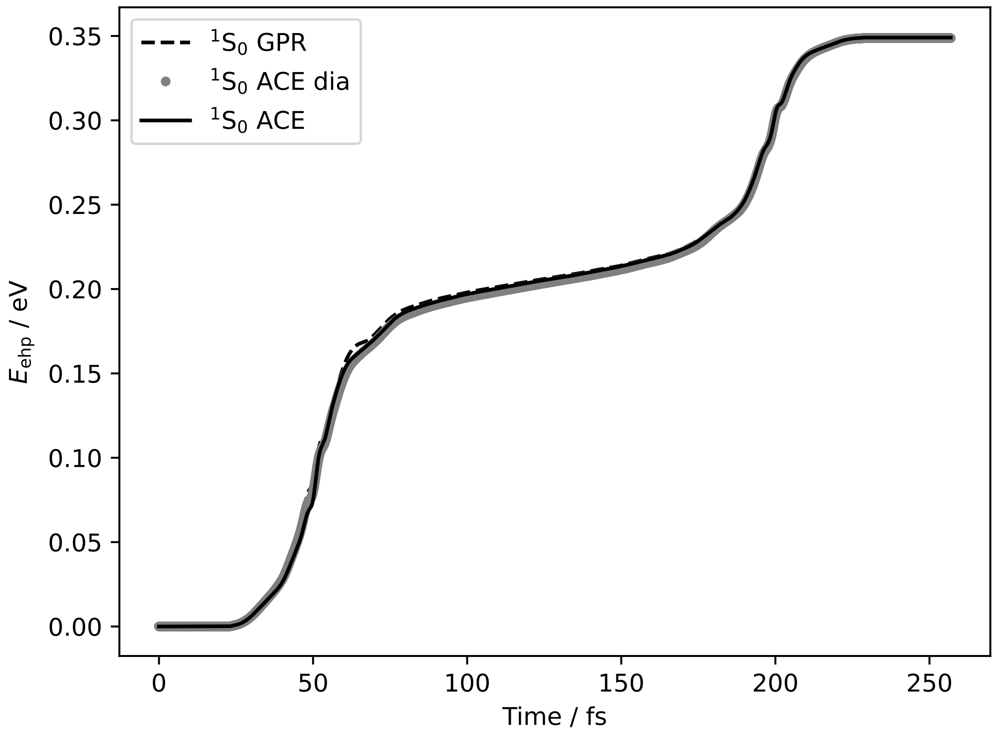

Figure S8: Accumulated energy loss over time (analogous to Fig. S5c) evaluated using the GPR model (dashed line), ACE model with diagonal elements (grey circles), and ACE model with all the elements of EFT (solid line).

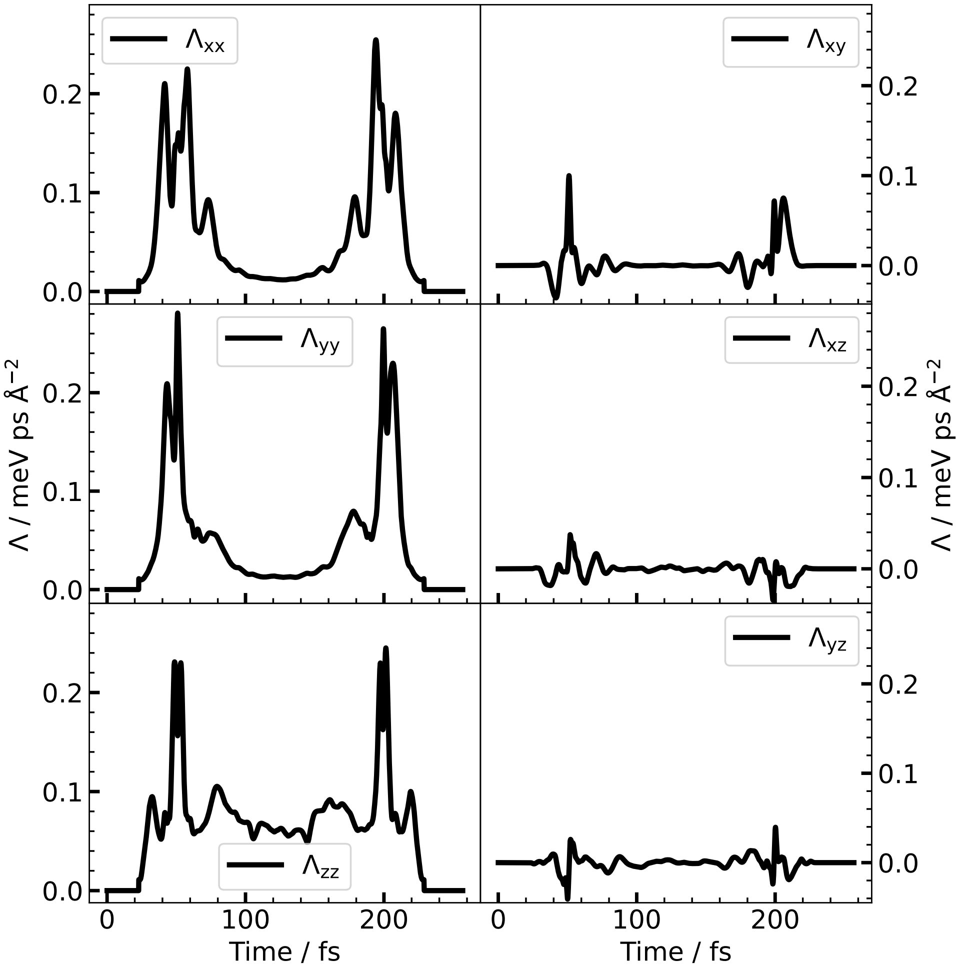

Figure S9: Different EFT elements along the scattering trajectory (same as in Fig. S5c) evaluated using the ${}^1S_0$ ACE model.

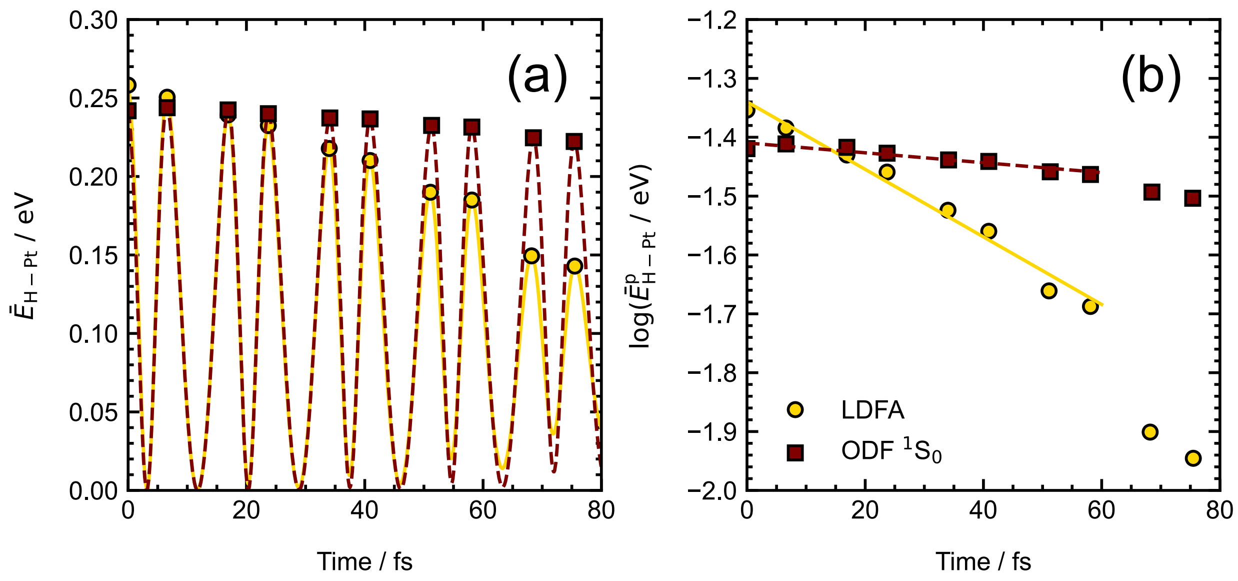

Figure S10: Average kinetic energy of the H-Pt symmetric stretch vibration, $\bar{E}_{\text{H-Pt}}$, plotted as a function of time. The dynamics are simulated by the EMT-LDFA and $^1S_0$ ODF models and the energy-time profile is plotted in panel (a). The points in panel (a) mark the amplitudes $\bar{E}^{\text{P}}_{\text{H-Pt}}$ which are used for the fit to extract the dynamical lifetime, shown in panel (b). Data points after 60 fs were excluded from the fit because the H atom would typically switch adsorption sites at this time.

decay along the H-Pt bond. A total of 1,000 simulations were performed for each LDFA and ODF, and the energy loss was averaged over those trajectories. Figure S10 shows the resulting energy-time profile. A decaying exponential $\bar{E}_{\text{H-Pt}} = \bar{E}^{\text{P}}_{\text{H-Pt}} e^{-kt}$ was fitted to the amplitudes, the inverse of the constant k corresponds to the lifetime $\tau^{\text{i}}_{q=0}$, where i $\in$ {LDFA, ODF}. We discarded data after 60 fs because the H atom would typically diffuse to neighbouring sites at this point. TableS3 compares the dynamic lifetimes detailed above to vibrational lifetimes calculated via the harmonic approximation using finite displacements—see Ref. 21 for details. This approach neglects anharmonic effects, but, in the case of ODF, we can evaluate friction at the perturbing frequency of the vibration and therefore do not have to make the zero-frequency (quasi-static) approximation as in MDEF. The corresponding ODF vibrational lifetime value therefore accounts for the quantum nature of the vibration (in the harmonic limit). Based on the excellent agreement between the vibrational lifetimes obtained via static and the dynamic methodology, we can conclude that the classical treatment of the hydrogen motion does not seem to be important for the determination of dynamical lifetimes for chemisorbed hydrogen on metal surfaces at room temperature.

Table S3: Comparison of vibrational lifetimes acquired dynamically via MDEF and statically via DFT-based electronic structure calculations.

Method	$\tau_{q=0}^{\mathrm{LDFA}}$ / ps	$\tau_{q=0}^{\mathrm{ODF}}$ / ps	τ^{exp}/ ps
Dynamic	0.17 ± 0.01	1.15 ± 0.2	-
Static-Harmonic	0.19	1.11	-
Exp.	-	-	0.8[20]

7 Sticking coefficients

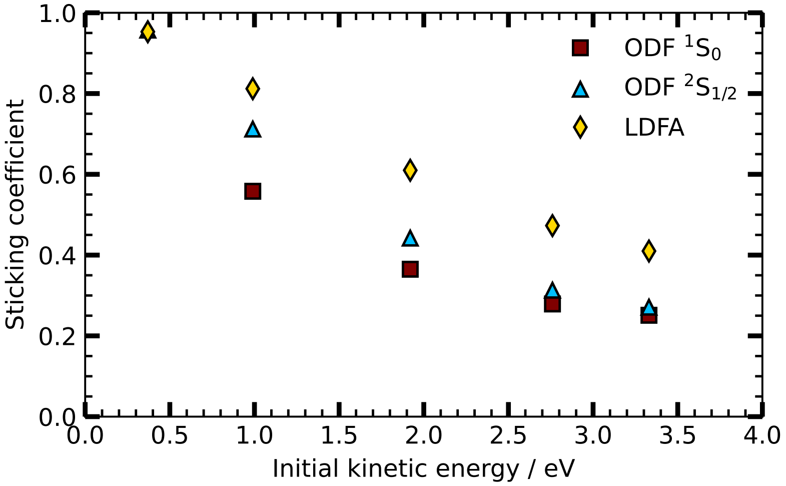

Figure S11: Sticking coefficients as a function of the initial kinetic energy from MDEF simulations with different friction models. The initial incidence angle is $\theta_{\mathrm{i}} = 45°$ and the H atoms lateral incidence direction was set to the $[10\bar{1}]$ direction. All initial conditions were chosen to be as close as possible to the experiment described in Ref. 22.

It is worthwhile to investigate how different friction theories affect the likelihood for adsorption. We launched MDEF simulations with the three friction models and varied the initial kinetic energy $E_{\mathrm{kin,i}}$. The fraction of adsorbed atoms are depicted in Figure S11. For $E_{\mathrm{kin,i}}$=0.37 eV, almost all H atoms remain at the surface. For the remaining initial kinetic energies, our LDFA values are in line with those reported for Ni(111), Ag(111) and Au(111).[22] Additionally, LDFA always gives larger sticking coefficients than ODF. This is consistent with the observation made by Spiering and Meyer for dissociative adsorption of H_2/Cu(111)[23] and N_2/Ru(0001),[24] albeit the differences between LDFA and ODF are more pronounced for our methodology. Furthermore, we note that

the differences between the singlet and the doublet ODF model get smaller with increasing kinetic energy. Unfortunately, there are currently no H atom sticking experiments where mono-energetic H atoms have been sent onto a metal surface to which these calculations could be compared. However, given the distinct differences between ODF and LDFA in terms of sticking verification of one of those models should be feasible through future experiments.

References

(1) Blum, V.; Gehrke, R.; Hanke, F.; Havu, P.; Havu, V.; Ren, X. G.; Reuter, K.; Scheffler, M. Ab initio molecular simulations with numeric atom-centered orbitals. *Computer Physics Communications* **2009**, *180*, 2175–2196.

(2) Lecroart, L.; Hertl, N.; Dorenkamp, Y.; Jiang, H.; Kitsopoulos, T. N.; Kandratsenka, A.; Bünermann, O.; Wodtke, A. M. Adsorbate modification of electronic nonadiabaticity: H atom scattering from p(2 × 2) O on Pt(111). *The Journal of Chemical Physics* **2021**, *155*, 034702.

(3) Monkhorst, H. J.; Pack, J. D. Special points for Brillouin-zone integrations. *Physical Review B* **1976**, *13*, 5188–5192.

(4) Van Lenthe, E.; Snijders, J. G.; Baerends, E. J. The zero-order regular approximation for relativistic effects: The effect of spin–orbit coupling in closed shell molecules. *The Journal of Chemical Physics* **1996**, *105*, 6505–6516.

(5) Perdew, J. P.; Burke, K.; Ernzerhof, M. Generalized Gradient Approximation Made Simple. *Physical Review Letters* **1996**, *77*, 3865–3868.

(6) Perdew, J. P.; Burke, K.; Ernzerhof, M. Generalized Gradient Approximation Made Simple. *Physical Review Letters* **1997**, *78*, 1396.

(7) Box, C. L.; Stark, W. G.; Maurer, R. J. Ab initio calculation of electron-phonon linewidths and molecular dynamics with electronic friction at metal surfaces with numeric atom-centred orbitals. *Electronic Structure* **2023**, *5*, 035005.

(8) Gardner, J.; Douglas-Gallardo, O. A.; Stark, W. G.; Westermayr, J.; Janke, S. M.; Habershon, S.; Maurer, R. J. NQCDynamics.jl: A Julia package for nonadiabatic quantum classical molecular dynamics in the condensed phase. *The Journal of Chemical Physics* **2022**, *156*, 174801.

(9) Auerbach, D. J.; Hertl, N.; Janke, S. M.; Kammler, M.; Kandratsenka, A.; Lecroart, L.; Wille, S. Molecular Dynamics Tian Xia 2 (MDT2): program for simulating the scattering of atoms and molecules from surfaces. Available at https://github.com/akandra/md_tian2. 2021; `https://github.com/akandra/md_tian2.git`.

(10) Kammler, M.; Janke, S. M.; Kandratsenka, A.; Wodtke, A. M. Genetic algorithm approach to global optimization of the full-dimensional potential energy surface for hydrogen atom at fcc-metal surfaces. *Chemical Physics Letters* **2017**, *683*, 286–290.

(11) Leimkuhler, B.; Matthews, C. Robust and efficient configurational molecular sampling via Langevin dynamics. *Journal of Chemical Physics* **2013**, *138*, 174102.

(12) Janke, S. M.; Auerbach, D. J.; Wodtke, A. M.; Kandratsenka, A. An accurate full-dimensional potential energy surface for H-Au(111): Importance of nonadiabatic electronic excitation in energy transfer and adsorption. *Journal of Chemical Physics* **2015**, *143*, 124708.

(13) Hertl, N.; Krüger, K.; Bünermann, O. Electronically Nonadiabatic H Atom Scattering from Low Miller Index Surfaces of Silver. *Langmuir* **2022**, *38*, 14162–14171.

(14) Deringer, V. L.; Bartók, A. P.; Bernstein, N.; Wilkins, D. M.; Ceriotti, M.; Csányi, G. Gaussian Process Regression for Materials and Molecules. *Chemical Reviews* **2021**, *121*, 10073–10141.

(15) Rasmussen, C. E.; Williams, C. K. I. *Gaussian Processes for Machine Learning, The MIT Press, United States*; 2005.

(16) Pedregosa, F. et al. Scikit-learn: Machine Learning in Python. *Journal of Machine Learning Research* **2011**, *12*, 2825–2830.

(17) Pearson, K.; Henrici, O. M. F. E. X. Contributions to the mathematical theory of evolution.—II. Skew variation in homogeneous material. *Philosophical Transactions of the Royal Society of London. (A.)* **1895**, *186*, 343–414.

(18) Sachs, M.; Stark, W. G.; Maurer, R. J.; Ortner, C. Equivariant Representation of Configuration-Dependent Friction Tensors in Langevin Heatbaths. `http://arxiv.org/abs/2407.13935`.

(19) Drautz, R. Atomic cluster expansion for accurate and transferable interatomic potentials. *99*, 014104.

(20) Paleček, D.; Tek, G.; Lan, J.; Iannuzzi, M.; Hamm, P. Characterization of the Platinum–Hydrogen Bond by Surface-Sensitive Time-Resolved Infrared Spectroscopy. *Journal of Physical Chemistry Letters.* **2018**, *9*, 1254–1259.

(21) Maurer, R. J.; Askerka, M.; Batista, V. S.; Tully, J. C. Ab initio tensorial electronic friction for molecules on metal surfaces: Nonadiabatic vibrational relaxation. *Phys. Rev. B* **2016**, *94*, 115432.

(22) Dorenkamp, Y.; Jiang, H. Y.; Kockert, H.; Hertl, N.; Kammler, M.; Janke, S. M.; Kandratsenka, A.; Wodtke, A. M.; Bunermann, O. Hydrogen collisions with transition metal surfaces: Universal electronically nonadiabatic adsorption. *Journal of Chemical Physics* **2018**, *148*, 034706.

(23) Spiering, P.; Meyer, J. Testing Electronic Friction Models: Vibrational De-excitation in Scat-

tering of H2 and D2 from Cu(111). *Journal of Physical Chemistry Letters* **2018**, *9*, 1803–1808.

(24) Spiering, P.; Shakouri, K.; Behler, J.; Kroes, G. J.; Meyer, J. Orbital-Dependent Electronic Friction Significantly Affects the Description of Reactive Scattering of N-2 from Ru(0001). *Journal of Physical Chemistry Letters* **2019**, *10*, 2957–2962.
